# Influencer Marketing in Ophthalmology

**DOI:** 10.22336/rjo.2025.24

**Published:** 2025

**Authors:** Consuela-Mădălina Gheorghe

**Affiliations:** Philologist, Certified translator

In an era where digital presence can significantly impact business success, ophthalmology practices and brands are increasingly turning to influencer marketing to expand their reach and establish trust with patients. While traditionally associated with fashion, beauty, or technology, influencer marketing is becoming a powerful tool in healthcare — especially in eye care — to educate, promote innovations, and drive patient awareness.

Another similar concept, derived from influencer marketing in ophthalmology, is online influencer marketing (OIM). It is recognized as a strategy in which an ophthalmological organization chooses and rewards online influencers to interact with their followers on social media to use the influencers’ special resources to market the organization’s products or services, thereby improving organization performance (Khang A, Jadhav B, Hajimahmud VA, Satpathy I. Synergy of AI and Fintech in the Digital Gig Economy. 1^st^ Edition, 2024, CRC Press, doi: https://doi.org/10.1201/9781032720104, e-ISSN: 9781032720104).

Online influencer marketing in ophthalmology is based on the following essential aspects: online influencers are chosen and compensated by organizations; influencers interact with their followers for financial gain, and organizations utilize influencers’ specialized resources to advertise products or services. Thus, these characteristics set online influencer marketing in ophthalmology apart from other marketing tactics, such as native advertising, customer review programs, viral marketing, celebrity endorsement, and seeding (Leung FF, Gu FF, Palmatier RW. Online influencer marketing. J. of the Acad. Mark. Sci. 2022;50:226-251. https://doi.org/10.1007/s11747-021-00829-4).

Influencers are people, groups of people, or even virtual personas who have amassed a following on social media and are considered to be digital opinion leaders with a sizable social following (Leung FF, Gu FF, Palmatier RW. Online influencer marketing. J. of the Acad. Mark. Sci. 2022;50:226-251. https://doi.org/10.1007/s11747-021-00829-4). A key distinction between influencers and celebrities is where they acquire their notoriety; yet, some influencers amass such a large following that they become internet celebrities. Influencers, who are not officially certified by any institutions, gain followers by actively sharing content on social media (Leung FF, Gu FF, Palmatier RW. Online influencer marketing. J. of the Acad. Mark. Sci. 2022;50:226-251. https://doi.org/10.1007/s11747-021-00829-4), in contrast to celebrities who have achieved success in a credentialed, institutional environment (such as acting, music, or sports).

Influencer marketing involves collaborating with individuals who have established credibility and a following on platforms like Instagram, YouTube, or TikTok to promote services or products (Khang A, Jadhav B, Hajimahmud VA, Satpathy I. Synergy of AI and Fintech in the Digital Gig Economy. 1st Edition, 2024, CRC Press, doi: https://doi.org/10.1201/9781032720104, e-ISSN: 9781032720104). In ophthalmology, this can include:

- **Promoting LASIK or cataract surgery outcomes;**
- **Showcasing new diagnostic or surgical technologies;**
- **Educating audiences on eye health and preventive care;**
- **Endorsing vision-related products (e.g., specialty contact lenses, dry eye therapies).**

Unlike traditional advertising, influencer marketing enables the creation of more authentic and engaging content, particularly when influencers share their personal experiences with eye care services.

In the ophthalmic space, influencers typically fall into four categories:

1. **Healthcare Professionals (HCPs):** Ophthalmologists and optometrists with a strong social media presence who share insights, patient stories, and clinical advancements.
2. **Patients:** Individuals who document their eye surgery or treatment journeys, particularly for procedures like LASIK or ICL implantation.
3. **Health & Wellness Influencers:** Content creators who focus on overall well-being and occasionally include eye health topics or partner with ophthalmic brands.
4. **Tech Influencers:** With the rise of wearable eye-tracking devices and digital vision tools, tech reviewers are becoming key players in the conversation.

Influencer marketing in ophthalmology is particularly effective in this medical field because it is based on trust and transparency, driven by education, and utilizes visual content that is well-received.

**Trust and Transparency:** Patients often find peer experiences more trustworthy than corporate messaging. Influencers can demystify procedures and share real-world results.

**Education-Driven:** Ophthalmology is a highly technical specialty. Influencers who explain conditions like glaucoma or keratoconus in layperson’s terms can empower patients.

**Visual Content Friendly:** Eye care lends itself well to striking visuals — from surgical animations to before-and-after images, perfect for platforms like Instagram or TikTok.

Until present, there have been many successful campaigns of influencer marketing in ophthalmology, such as:

- **LASIK Campaigns:** Clinics collaborate with lifestyle influencers who undergo LASIK and share their journey, boosting awareness and interest in corrective eye surgery.
- **Dry Eye Awareness Month:** Eye care brands partner with optometrists on YouTube and Instagram to educate about symptoms, treatment options, and innovations.
- **Smart Eyewear Launches:** Influencers showcase smart glasses or blue-light blocking lenses, highlighting their benefits for digital eye strain.

Regarding the ethical guidelines, ophthalmology, just like healthcare influencer marketing, must navigate regulatory and ethical considerations, including:

- **Disclosure Compliance:** All partnerships must be disclosed in accordance with FTC guidelines (https://www.ftc.gov/business-guidance/advertising-marketing/endorsements-influencers-reviews).
- **Accuracy of Information:** Content must be evidence-based and not misleading.
- **HIPAA Considerations:** Patient influencers must provide explicit consent when sharing medical information or outcomes (https://www.hhs.gov/hipaa/for-professionals/security/laws-regulations/index.html).

As far as the future of influencer marketing in ophthalmology is concerned, as younger, digitally native patients increasingly turn to social media for health information, influencer marketing will become a staple in ophthalmic practice marketing strategies. Collaborations with credible voices can not only attract new patients but also build long-term trust and brand loyalty.

In conclusion, ophthalmology professionals and organizations embracing this trend should prioritize authenticity, education, and ethical transparency, ensuring that their influencer strategies deliver genuine value to the public while advancing the mission of promoting better eye health.

Assist. Prof. Consuela-Mădălina Gheorghe, PhD, Philologist, Certified translator

